# Temporal Patterns of Skin-Fixed Depth Electrodes During Stereotactic Electroencephalography Recording

**DOI:** 10.7759/cureus.104348

**Published:** 2026-02-26

**Authors:** Takayuki Kikuchi, Masahiro Sawada, Ai Demura, Yukihiro Yamao, Daisuke Yamada, Takeshi Yoshida, Katsuya Kobayashi, Akio Ikeda, Riki Matsumoto, Yoshiki Arakawa

**Affiliations:** 1 Department of Neurosurgery, Kyoto University Graduate School of Medicine, Kyoto, JPN; 2 Department of Neurosurgery, Kyoto University Hospital, Kyoto, JPN; 3 Department of Pediatrics, Kyoto University Graduate School of Medicine, Kyoto, JPN; 4 Department of Neurology, Kyoto University Graduate School of Medicine, Kyoto, JPN; 5 Department of Epilepsy, Movement Disorders and Physiology, Kyoto University Graduate School of Medicine, Kyoto, JPN

**Keywords:** cone-beam computed tomography, electrode dislocation, epilepsy surgery, skin swelling, stereotactic electroencephalography

## Abstract

Introduction: Stereotactic electroencephalography (SEEG) is increasingly used to evaluate patients with refractory epilepsy. While anchor bolts are widely used for depth electrode fixation, their lack of insurance coverage or technical limitations may necessitate alternative methods, such as fixation to the skin. Few studies have investigated how fixation methods affect electrode dislocation during recording.

Methods: To clarify the time-dependent pattern of skin-fixed electrode dislocation, depth electrode dislocation during recording was evaluated in 41 electrodes from four patients with anchor bolt fixation and 66 electrodes from five patients with skin fixation.

Results: Twenty-nine electrodes in the anchor bolt group and 45 electrodes in the skin fixation group were targeted to the anterior temporal or perisylvian regions. The mean cumulative migration in longitudinal direction during recording was 4.34 ± 2.43 mm in the skin fixation group compared to 0.16 ± 0.43 mm in the anchor bolt group (p < 0.0001). In the skin fixation group, the net migration during recording was -2.53 ± 2.20 mm, and the net migration from the original position was +0.37 ± 1.87 mm in the skin fixation group (negative values represent inward migration). Electrode migration was most prominent during the bilateral implantation procedure (+6.01 ± 3.48 mm). The mean electrode shift (lateral displacement) in the skin fixation group was 0.87 ± 0.68 mm. No migration-related complications were observed.

Conclusion: Temporal pattern of skin-fixed depth electrode dislocation was evaluated in detail. Fixation to the skin carries a higher risk of electrode migration compared to anchor bolts. Although this rarely causes clinical complications, caution is warranted both for risk prevention and when interpreting anatomo-electrophysiological relationships.

## Introduction

Stereotactic electroencephalography (SEEG) is an invasive method used for evaluating the epileptogenic network in refractory epilepsy [1]. The procedure is gaining popularity because it is safe, minimally invasive, efficiently estimates the epileptogenic network, and provides effective seizure control following resection [2-5]. However, the availability of SEEG-specific devices varies by country, and the use of modern instruments often depends on economic and socio-medical factors. In addition, anchor bolts cannot be used in children with thin skulls, making skin fixation the preferred option [6]. In Japan, where anchor bolts have not yet been approved, various fixation methods have been proposed, but most facilities fix the electrode to the skin [7-10]. Skin-fixed electrodes may be more susceptible to displacement due to surgical site swelling or intraoperative head positioning compared to anchor bolt fixation [7]. Such instability could influence the interpretation of electrocorticography, functional mapping, and the risk of hemorrhage or infection. This study aims to characterize the temporal patterns of electrode dislocation during SEEG recording with skin fixation, with a primary focus on changes over time and their implications for patient management during the recording period.

## Materials and methods

This study was approved by the Institutional Review Board (YC1192, R2088). Informed consent was obtained from patients who underwent anchor bolt fixation (YC1192). The requirement for individual informed consent was waived for those who underwent skin fixation (R2088).

Patient population

Patients with refractory focal epilepsy who underwent SEEG recording between January 2017 and September 2024 were included in this study. All patients underwent a comprehensive non-invasive presurgical evaluation, including long-term video-EEG monitoring, MRI, fluorodeoxyglucose positron emission tomography (FDG-PET), neuropsychological assessment, and magnetoencephalography (MEG), and were diagnosed as having focal epilepsy and their seizures were refractory [11]. All patients were suspected of having bilateral or deep-seated epileptogenic areas.

Implantation procedure

**Figure 1:**
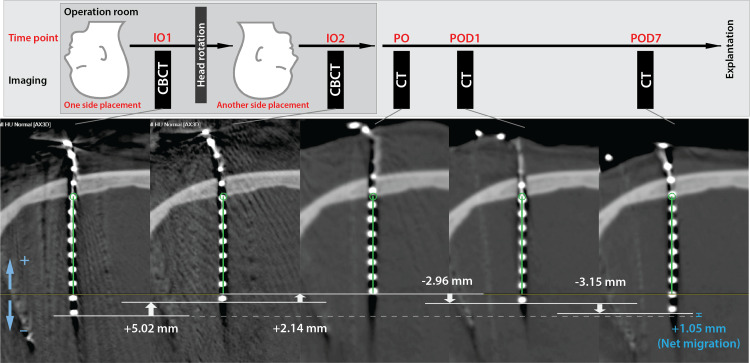
Study protocol and images from a representative patient who underwent bilateral stereotactic electrode implantation Upper low: Migration distance of each interval was used for analysis. For bilateral implantation, intraoperative CBCT was performed twice, once after implantation in each hemisphere. Lower low: Position of the depth electrode targeting the right parahippocampal gyrus at each time point. White arrows indicate migration distance. Negative values indicate electrode advancement (inward migration). CBCT: cone beam computed tomography; IO1: intraoperative image 1; IO2: intraoperative image 2; POD: postoperative day; PO: post-implantation

Intracranial depth electrodes with a diameter of 0.86 mm or 1.12 mm (Ad-Tech Medical Instrument Corporation, Wisconsin, USA) or those with a diameter of 1.5 mm (Unique Medical, Tokyo, Japan) were implanted using the Leksell G-frame system (Electa AB, Stockholm, Sweden) after trajectory planning using surgical planning software (iPlan or Elements, Brainlab AG, Munich, Germany). The electrodes were implanted orthogonally using a twist drill [1]. In the first two years, the electrodes were implanted using 2.4 mm diameter drill bit kits and obturators and fixed with anchor bolts (Ad-Tech Medical Instrument Corporation, Wisconsin, USA) [12]. In subsequent years, electrodes were fixed on the skin with sutures. The bone was perforated using a 3.2 mm diameter Salcman twist drill, and an electrode was introduced with a 2.1 mm insertion canula kit (Electa AB, Stockholm, Sweden). Depth electrodes made by Unique Medical were used in the skin fixation group, whereas Ad-tech electrodes were implanted in the anchor-bolt fixation group. The implantation procedures were performed in the supine position with the head turned to the contralateral side of implantation. For bilateral cases, the head was rotated 180 degrees intraoperatively after the implantation of the first hemisphere was completed (Figure 1).

Evaluation of electrode position

Computed tomography (CT) scan was performed post-implantation (PO) immediately, on postoperative day 1 (POD1), and on POD 6-7 (POD7) to confirm electrode locations. Intraoperative cone beam CT (CBCT) images, after the placement of electrodes for each hemisphere, were also used for analysis; two CBCT images (IO1 and IO2) were obtained from patients with bilateral implantation, and one CBCT image (IO2) was obtained from patients with unilateral implantation (Figure 1). All images were integrated into the stereotactic planning software (Elements, Brainlab AG) and registered with each other. Dislocation was measured for each interval. Electrode migration (longitudinal axis) and shift (lateral displacement) were measured. Deeper displacement of the electrode was calculated as a negative value. For comparison between the skin-fixation group and anchor bolt fixation group, cumulative and net migration during recording were calculated as the sum of absolute values and the simple algebraic sum of postoperative migration (PO to POD1 and POD1 to POD7), respectively. Net migration from original position (IO1 to IO2 (bilateral case only) + IO2 to PO + PO to POD1 + POD1 to POD7) was also evaluated in the skin fixation group. Electrode shift was calculated using the pooled data from all postoperative observation intervals (PO to POD1 and POD1 to POD7). A correlation analysis between axial and perpendicular electrode movements was also performed. The distance was presented as the mean and standard deviation (SD). JMP Pro 15 (SAS Institute Inc., Cary, NC, USA) was used for statistical analysis. Statistical significance was set at P = 0.05.

## Results

Patient demographics

Eleven patients with refractory epilepsy underwent stereotactic depth electrode implantation. One patient in the skin fixation group was excluded from further analysis because only two depth electrodes were implanted, targeting the bilateral hippocampi through conventional burr holes prepared in the occipital areas, rather than using an orthogonal trajectory. Twenty-nine (70.7%) electrodes in the anchor bolt group and 45 (68.2%) electrodes in the skin fixation group were targeted to the anterior temporal or perisylvian regions, whose trajectories typically traverse the temporal muscle. Five electrodes that required adjustment based on intraoperative CBCT results were excluded. Overall, 41 electrodes from four patients with anchor-bolt fixation and 61 electrodes from six patients with skin fixation were analyzed. Details of the patients and procedures are summarized in Table 1.

**Table 1:** Patient demographics

Fixation group	Anchor bolt	Skin	Total
Subjects (N)	4	6	10
Mean age (y)	25.5	27.5	26.7
Sex (F:M)	3:1	4:2	7:3
Bilateral implantation	2	2	4
Implanted depth electrodes (N)	41	66	107
Left: Right	16:25	34:32	50:57
Target area	N (%)	N (%)	N (%)
Temporal anterior	19 (46.3)	24 (36.4)	43 (40.2)
Perisylvian	10 (24.4)	21 (31.8)	31 (29.0)
Temporal posterior	1 (2.4)	5 (7.6)	6 (5.6)
Frontal ventral	1 (2.4)	3 (4.5)	4 (3.7)
Frontal dorsal	6 (14.6)	3 (4.5)	9 (8.4)
Parietal	4 (9.8)	8 (12.1)	12 (11.2)
Occipital	0 (0)	2 (3.0)	2 (1.9)

Intracranial recording and functional mapping were performed in all patients to successfully identify the seizure onset network. One patient in the anchor-bolt fixation group experienced a transient headache caused by intraventricular hemorrhage during the explantation procedure. No complications were observed in the skin fixation group.

Electrode dislocation

**Figure 2:**
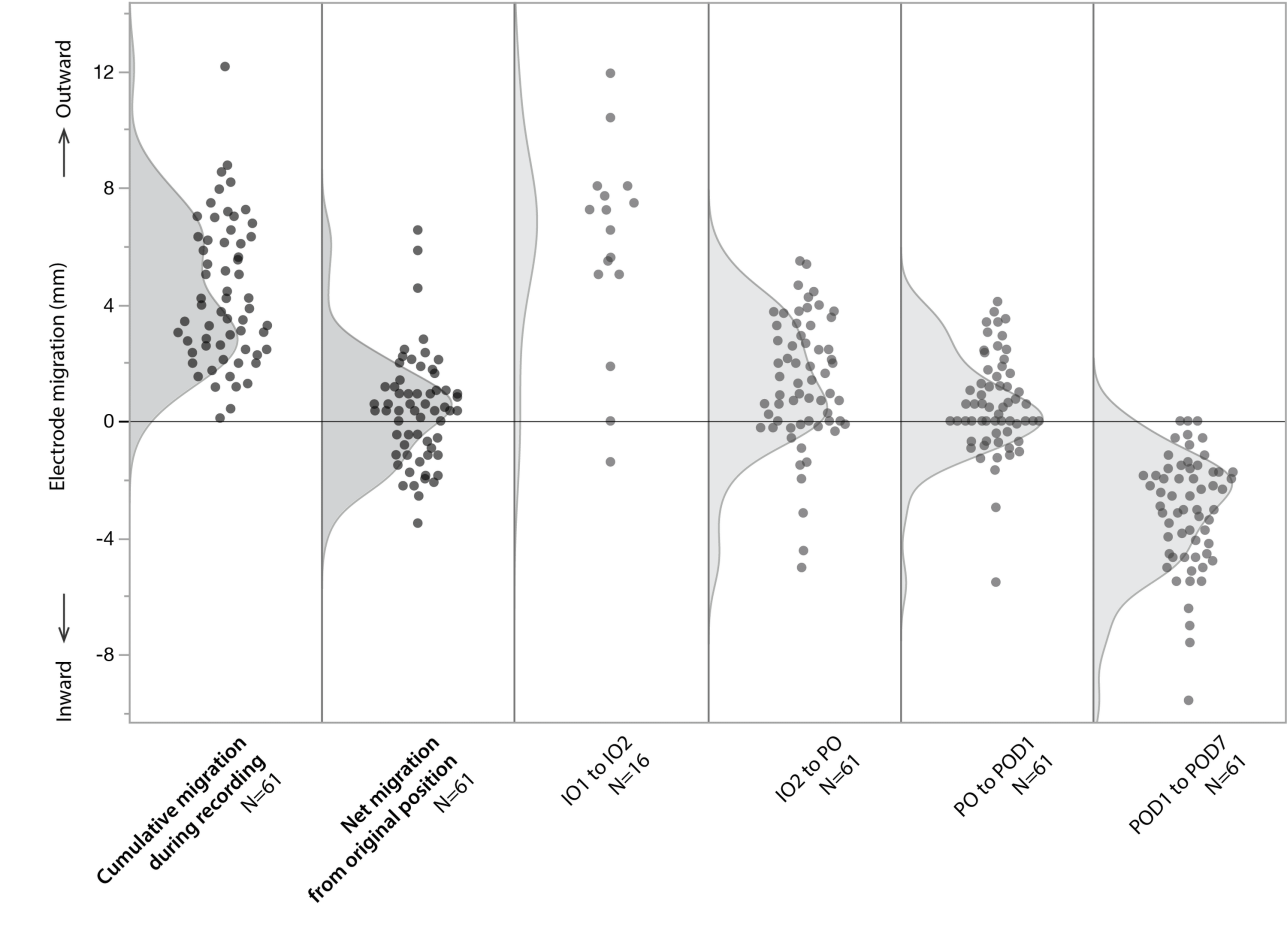
Details of electrode migration in the skin-fixation group Each gray marker indicates the migration distance of an electrode in each interval. The gray shaded area represents a histogram of the values. IO1, IO2, PO, POD1, and POD7 indicate the timing of evaluation. IO1: intraoperative image 1; IO2: intraoperative image 2; POD: postoperative day; PO: post-implantation

The mean cumulative migration during recording was 4.34 ± 2.43 mm in the skin fixation group compared to 0.16 ± 0.43 mm in the anchor bolt fixation group (p < 0.0001). Mean net migration during recording was -2.53 ± 2.20 mm in the skin fixation group compared to 0.13 ± 0.44 mm in the anchor bolt fixation group (p < 0.0001). The mean net migration from the original position in the skin fixation group was +0.37 ± 1.87mm. The mean electrode migration in each interval was +6.01 ± 3.48 mm from IO1 to IO2, +1.33 ±2.19 mm from IO2 to PO, +0.56 ± 1.68 mm from PO to POD1, and -3.09 ± 1.94 mm from POD1 to POD7 (Figures 1, 2).

**Figure 3:**
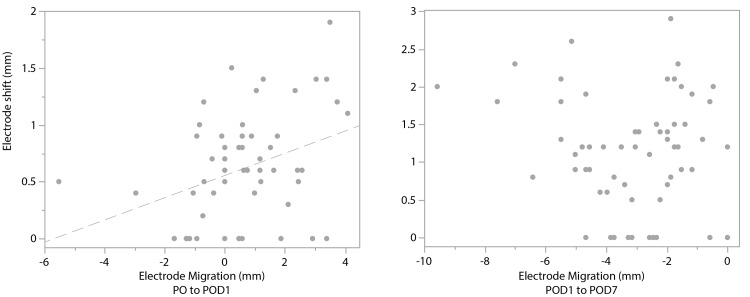
Electrode shift and migration in the skin-fixation group Scatter plots of migration and shift of depth electrodes during recording. POD: postoperative day; PO: post-implantation

The mean shift in the skin fixation and the anchor-bolt fixation groups was 0.87 ± 0.68 mm and 0.21 ± 0.44 mm, respectively (p < 0.0001). In the correlation analysis between migration and shift (Figure 3), a weak positive correlation (r = 0.34, P = 0.0075) was demonstrated during the PO-POD1 interval. In contrast, the POD1-POD7 interval showed a weak negative correlation that did not reach statistical significance (r= -0.15, P = 0.2488).

## Discussion

Trend of electrode dislocation

Our study found a mean net electrode migration of -2.53 mm during recording. Yindeedej et al. reported the mean “tip shift distance” as 1.4 mm (inward migration) during the mean implantation period of 8.1 days [7]. They and other groups suggested that the migration was associated with changes in conditions of the skin and subcutaneous tissue [7,9]. The incision of the skin and drilling of the skull easily facilitates the swelling of these tissues. This swelling leads to outward migration of the electrodes. Generally, skin swelling worsened over the first few postoperative days and improved within one week [13]. In our study, electrodes migrate outward in the intraoperative or early postoperative period and advance in the later period, and the most prominent migration was intraoperative outward migration (Figure 2). The greater migration in our study may be attributed to differences in the implantation procedures. Our implantation procedure needs head rotation in bilaterally implanted patients, which may amplify surgical site swelling due to gravity. Yindeedej et al. used a frameless robot system that generally does not require head rotation. We assume that the migration along the trajectory also induces displacement in the perpendicular direction, due to factors such as the orientation of white matter fibers. Our result failed to demonstrate a clear correlation between migration along the trajectory and perpendicular shift, although a weak tendency for larger inward or outward migration with larger perpendicular shift exists. There may be other factors, such as interval length and manufacturing-related straightness deviation of electrodes.

Safety considerations

While skin fixation leads to greater migration, the associated risks appear manageable, as no migration-related complications were observed. Furthermore, 64% of electrode (39 out of 61) did not advance beyond their original position (net migration in Figure 2). It may be reasonable to assume that the risk is low as long as the electrode does not advance beyond its original position.

Gradual electrode migration over time may influence the anatomical interpretation of electrophysiological findings, particularly as the interval from imaging increases. Because the implantation period is inherently limited and seizure occurrence cannot be predicted in advance, electrophysiological data obtained throughout the implantation period are clinically valuable. For this reason, maintaining anatomical reliability throughout the recording period is important. It might be recommended to confirm the position of the electrodes on a regular basis using CT or magnetic resonance imaging, although it harbors the risk of seizure during imaging and heat-induced brain injury. We performed three CT scans during electrode implantation from the perspective of patient safety and risk prevention. However, this protocol results in higher radiation exposure compared with standard protocols. The optimal imaging protocol should be carefully considered to balance patient safety and radiation exposure. Clinicians should also consider that a shift of one contact can occur during recording when interpreting findings of electrocorticography or functional mapping.

An alternative approach to preventing electrode migration is securing the electrode to the skull. However, this remains challenging in Japan, as it requires the use of anchor bolts. Other skull fixation techniques have been proposed, such as exposing the skull through a large skin incision or a minimal skin incision for direct fixation [9,10]. These methods may be viable options, but the invasiveness and procedural complexity must be carefully weighed.

Limitations

This was a retrospective study using a small cohort. The results may be influenced by bias. Different electrodes were used in the anchor-bolt fixation (Ad-Tech) and the skin fixation group (Unique Medical). We used different implantation devices in each group, which may have influenced the study results. However, since this study focused on electrode movement during the recording period after the implantation procedure, we believe that the impact of this difference is minimal. Although the cohort included early cases of SEEG recording and all implantation procedures were performed by a single operator (T.K.), the procedures in the skin fixation group, which showed significantly greater migration, were conducted in the later part of the study. Skin swelling was not assessed because imaging was not optimal for direct evaluation of skin thickness.

## Conclusions

This study indicates the higher propensity of electrode migration with the skin fixation method compared with the anchor-bolt fixation method, possibly related to skin swelling. The electrode with skin fixation initially migrates outward and subsequently moves inward toward its original position. Although SEEG recording can be safely performed using skin fixation, caution is warranted when interpreting the anatomical relationships of electrophysiological findings, as electrode migration may alter the spatial relationship between recording sites and anatomical structures.
